# Development of a patient-reported outcome (PRO) measure to assess patient perceptions of simplicity and complexity of treatment for type 2 diabetes

**DOI:** 10.1186/s41687-023-00614-7

**Published:** 2023-09-06

**Authors:** Katie D. Stewart, Louis S. Matza, Hiren Patel, Kristina S. Boye

**Affiliations:** 1 Evidera, Bethesda, MD USA; 2 Eli Lilly and Company, Indianapolis, IN USA

**Keywords:** Type 2 diabetes, Patient-reported outcome measure, PRO, Concept elicitation, Qualitative research

## Abstract

**Introduction:**

Treatments for type 2 diabetes vary widely in their complexity. The simplicity or complexity of a treatment regimen may have an impact on patient preference, treatment adherence, and health outcomes. The purpose of this qualitative study was to develop two draft patient-reported outcome instruments focusing on patients’ experience with simplicity and complexity of treatment for type 2 diabetes.

**Methods:**

The instruments were developed in a series of steps: gather information to support development of a concept elicitation interview guide (literature review and expert interviews), concept elicitation interviews with patients (N = 30), cognitive interviews with patients (N = 20), and a translatability assessment.

**Results:**

In concept elicitation interviews, patients with type 2 diabetes reported a range of treatment attributes that influence their perceptions of treatment simplicity and complexity, such as injection devices, medication preparation, dose timing, dose frequency, ease of taking the correct dose, flexibility of dose schedule, remembering to take medication, and food requirements. Two draft questionnaires were developed based on the literature review, expert interviews, and concept elicitation interviews with patients. Revisions were made to these draft instruments based on qualitative interviews with patients and translatability assessment.

**Discussion:**

The qualitative research conducted in this study supports the content validity of two newly developed instruments, the Simplicity of Diabetes Treatment Questionnaire (Sim-Q) and the Simplicity of Diabetes Treatment Questionnaire-Comparison (Sim-Q-Comp), designed to assess the simplicity and complexity of diabetes treatment from the patient’s perspective.

**Supplementary Information:**

The online version contains supplementary material available at 10.1186/s41687-023-00614-7.

## Plain language summary

Treatments for type 2 diabetes vary widely in their complexity, and previous research suggests that simpler treatments may have benefits for patients, such as better medication adherence and improved glycemic control. Despite the benefits of treatment simplicity, there are limited options for assessing simplicity of treatment from the patient perspective. This study was designed to develop two patient-reported outcome measures that assess simplicity and complexity of treatment for type 2 diabetes. Thirty patients with type 2 diabetes reported a range of treatment attributes that influence their perceptions of treatment simplicity and complexity. These attributes included injection devices, medication preparation, dose timing, dose frequency, ease of taking the correct dose, flexibility of dose schedule, and food requirements. Two questionnaires were developed based on literature review, expert interviews, and patient interviews (one questionnaire for rating a single treatment, and another questionnaire for comparing two treatments). Revisions were made to the draft instruments based on feedback from 20 additional participants and a translatability assessment. The resulting instruments are called the Simplicity of Diabetes Treatment Questionnaire (Sim-Q) and Simplicity of Diabetes Treatment Questionnaire-Comparison (Sim-Q-Comp). Future research with more patients is needed to further examine the psychometric properties of the questionnaires.

**Supplementary Information:**

The online version contains supplementary material available at 10.1186/s41687-023-00614-7.

## Introduction

Treatments for type 2 diabetes vary widely in their complexity. While relatively simple treatment regimens may involve once-daily oral treatment [1] or once-weekly injections [2–4], more complex regimens include polytherapy and multiple daily injections linked to mealtimes [5–7]. Adding to the complexity of overall diabetes management, patients may be required to perform additional tasks such as glucose monitoring or injection device preparation [8, 9].

Previous research has suggested that simpler treatment regimens may have a range of benefits for patients. Patients with type 2 diabetes have consistently been found to prefer treatment regimens that they perceived to be simpler with attributes such as reduced dose frequency, fewer restrictions associated with dose timing, fewer food-related requirements, and less complex injection devices [10–14]. In patients with type 2 diabetes, greater treatment simplicity has been found to be associated with better medication adherence [15, 16] and improved glycemic control [15, 17].

Despite the benefits of treatment simplicity, there are limited options for assessing treatment simplicity from the patient perspective. Patient-reported outcome (PRO) measures can provide information on the impact of disease and treatment from the patient’s perspective, and the importance of this information is becoming more commonly recognized by clinicians and regulatory agencies [18]. Some questionnaires have been designed to assess concepts that may be related to treatment simplicity, such as treatment burden (e.g., the Treatment-Related Impact Measures for Diabetes and Devices, Diabetes Medication Satisfaction, Diabetic Treatment Burden Questionnaire) [19–21] and treatment satisfaction (e.g., Insulin Treatment Satisfaction Questionnaire; Diabetes Treatment Satisfaction Questionnaire, Patient Satisfaction with Insulin Treatment) [20, 22–24]. In addition, questionnaires focusing on injectable treatment have included items assessing ease of use, injection devices, and ease of taking an injectable medication [24, 25]. However, no known PRO measures have been developed to assess simplicity or complexity of treatment for type 2 diabetes.

Therefore, this qualitative study was designed to support the development of two PRO measures that assess simplicity and complexity of treatment for type 2 diabetes. The first questionnaire was designed to assess perceptions of simplicity/complexity of a current treatment, while the second questionnaire asks patients to compare the simplicity/complexity of their current treatment with a previous treatment. First, concept elicitation interviews were conducted with patients to identify aspects of medication treatment for type 2 diabetes that they perceived to be simple or complex. Results informed the development of the draft questionnaires, which were refined based on feedback from patients who completed the instruments. This project was designed in accordance with Food and Drug Administration (FDA) and ISPOR preferences and recommendations for qualitative research conducted to inform PRO instrument development [26–30].

## Methods

### Overview of study steps

**Fig. 1:**
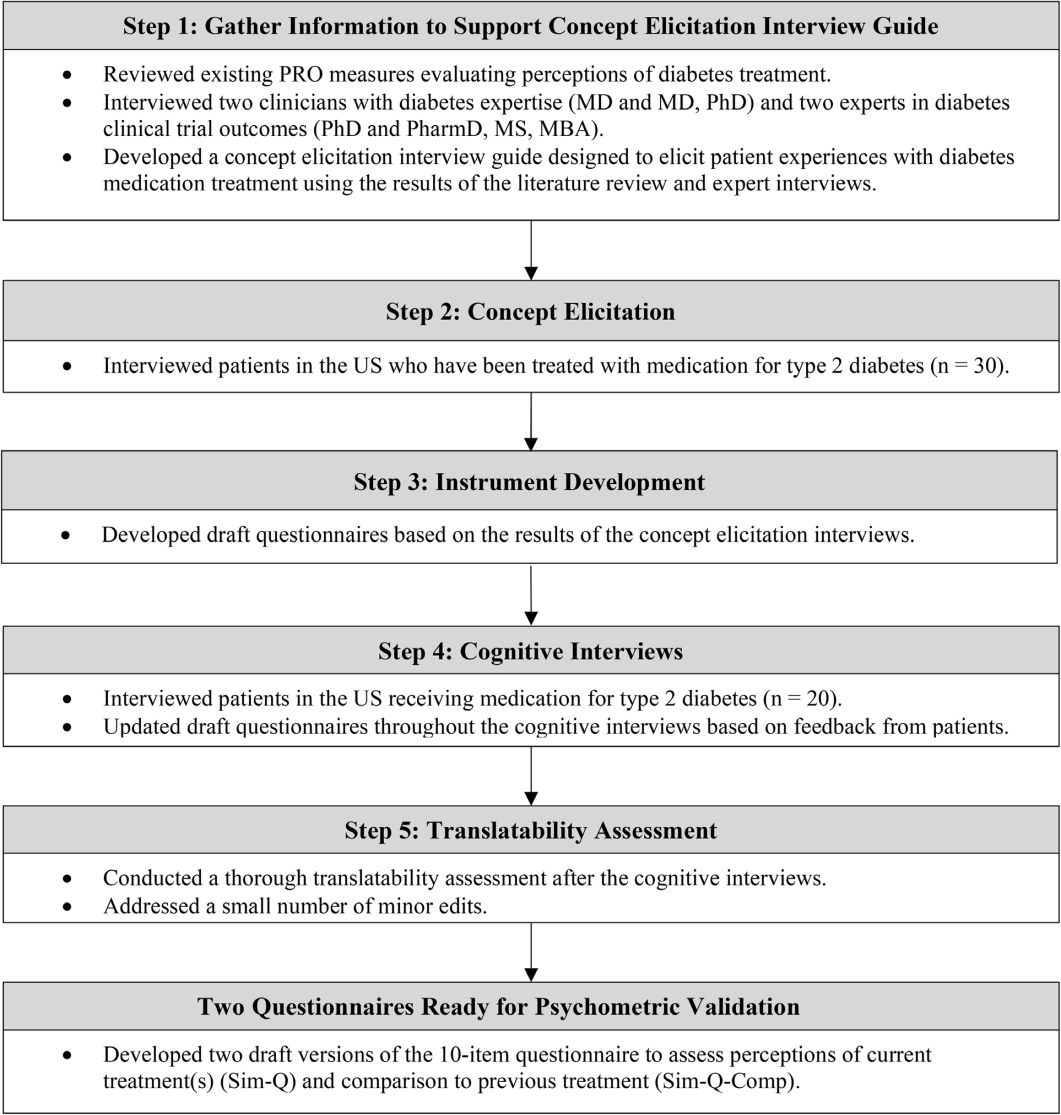
Summary of instrument development of the Sim-Q and the Sim-Q-Comp

The Simplicity of Diabetes Treatment Questionnaire (Sim-Q) and the Simplicity of Diabetes Treatment Questionnaire-Comparison (Sim-Q-Comp) were developed through a series of five steps summarized in Fig. 1. First, background information was gathered to inform the development of the qualitative interview guide for the concept elicitation interviews. Existing PROs assessing perceptions of diabetes treatments were reviewed to identify concepts relevant to simplicity and complexity of diabetes treatment. In addition, interviews were conducted with two diabetes clinical experts (MD and MD/PhD) and two experts in diabetes clinical trial outcomes (PhD and PharmD, MS, MBA). These interviews focused on identifying aspects of diabetes treatment that may influence patient perceptions of treatment simplicity or complexity. The results of these interviews and the literature review informed the development of a concept elicitation interview guide.

In step 2, concept elicitation interviews were conducted with patients being treated with medication for type 2 diabetes in the US. Interviews focused on identifying aspects of medication treatment that patients commonly perceive as either simple or complex. Results of these qualitative interviews were used to develop two draft patient-reported questionnaires (step 3). The *status version* of the questionnaire was designed to evaluate simplicity and complexity of current medication treatment for type 2 diabetes. A parallel *comparison version* was designed to compare current treatment with previous treatment.

In step 4, the draft questionnaires were evaluated in cognitive interviews with patients receiving treatment for type 2 diabetes in the US. Participants were asked to complete the draft status questionnaires thinking about their current treatment for type 2 diabetes, and to complete the comparison version of the questionnaire comparing their current treatment with a previous treatment. The interview focused on how each respondent understood and responded to the questionnaire, with detailed discussion on interpretation of the instructions, items, and response options. The draft questionnaires were updated throughout the cognitive interviews based on feedback from participants and further refined based on a translatability assessment (step 5). The two draft questionnaires resulting from this process are called the Simplicity of Diabetes Treatment Questionnaire (Sim-Q) and the Simplicity of Diabetes Treatment Questionnaire-Comparison (Sim-Q-Comp).

All study methods and materials were approved by an independent review board (Ethical and Independent Review Services [E&I] study numbers 20103 and 21001). All participants provided informed consent prior to engaging in study procedures. Interviews were audio recorded and transcribed for coding and qualitative analysis.

### Participants

Qualitative interviews were conducted with patients during the concept elicitation and cognitive interview phases (steps 2 and 4). All participants were required to be (1) diagnosed with type 2 diabetes by a recognized medical professional for at least 6 months; (2) ≥ 18 years of age; (3) currently treated with medication for type 2 diabetes for at least 3 months; (4) residing in the US; (5) able to speak, read, and understand English; (6) able and willing to give informed consent prior to study entry; and (7) able to complete the protocol requirements. Potential participants were excluded if they (1) had a cognitive impairment, hearing difficulty, visual impairment, acute psychopathology, or insufficient knowledge of the English language that, in the opinion of the clinical site investigator or project staff screener, could interfere with their ability to provide consent and complete the interview; (2) were diagnosed with type 1 diabetes, latent autoimmune diabetes, or gestational diabetes; or (3) were employed by a pharmaceutical company or had a direct role in treating patients with diabetes.

Patients with type 2 diabetes were identified from each clinic’s medical records. A member of the clinic staff approached potentially eligible patients during a usual clinical visit or via telephone to introduce the study and assess interest in participation. Efforts were made to obtain a clinically broad sample, including patients treated only with oral medication and patients treated with injectable medication to ensure the sample was diverse with regard to complexity of their current treatment regimen. Participants for both phases were recruited from four clinical sites in four states across the US (KY, GA, PA, FL), including two research facilities, one endocrinology specialist, and one family medicine practice. Clinical sites were selected based on geographic diversity, interest in assisting with qualitative research, and ability to recruit eligible participants.

### Data collection

#### Concept elicitation (step 2)

Concept elicitation interviews were conducted by three researchers trained in qualitative interviewing. All interviews were conducted by telephone according to a semi-structured interview guide designed to elicit discussion of patients’ experience with treatment for type 2 diabetes, with a focus on aspects of treatment perceived to be simple or complex. The interviews were designed and scheduled to last approximately 60–90 min. Each interview began with open-ended discussion of the medication treatment process (e.g., Could you please describe the process for taking your medication? How do you take this medication?) and the glucose monitoring process (if applicable). Discussion continued with questions about the aspects of the treatment that make it simple or complex (e.g., What about your treatment is simple or easy? What about your treatment is complex or difficult?), the impact of treatment on the participant’s life (e.g., How does your treatment of diabetes impact your life? Does the complexity of this treatment have an impact on your life?), and how simplifying treatment may impact the participant’s life (e.g., How might the impact be reduced if treatment were simplified?). The guide included probes for specific aspects of medication treatment that may contribute to simplicity and complexity such as the medication process, dose frequency, dose timing, food requirements, flexibility, and monitoring of blood sugar.

#### Cognitive interviews (step 4)

Cognitive interviews were conducted to evaluate the content validity of the draft questionnaires in terms of ease of use, clarity, comprehensibility, comprehensiveness, redundancy, and relevance. At the time of each interview, participants first completed the draft Sim-Q and Sim-Q-Comp. Then, cognitive interviews were conducted according to a semi-structured guide to evaluate participants’ understanding of the instructions, items, and response options. Each participant was asked to describe their interpretation of the questionnaire instructions, their interpretation of each item, and how they selected a response.

Participants from Step 2 were not excluded from the cognitive interviews in Step 4, but the study team attempted to minimize re-use of concept elicitation participants in the cognitive interviews. Only two participants from the concept elicitation phase were also included in the cognitive interviews.

### Measures

Each participant completed a brief sociodemographic form including items on age, gender, living situation, employment, education level, racial/ethnic background, and general health-related questions. For each participant, a clinical information form was completed by site personnel. This form included questions about the duration of type 2 diabetes, current medications for treatment of type 2 diabetes, the most recent HbA1c value, and the patient’s height and weight to calculate body mass index.

### Analysis procedures

#### Quantitative analysis

Responses on the patient-completed and site-completed forms were summarized with descriptive statistics (means and standard deviations [SDs] for continuous variables; frequencies and percentages for categorical variables). No statistical comparisons were conducted.

#### Qualitative analysis

Qualitative data from the concept elicitation and cognitive interviews were coded using a content analysis approach using ATLAS.ti software [31, 32]. An analytic coding dictionary of themes, concepts, and terms was developed based on the interview guide, and revised as needed during coding to capture emerging concepts. The coding dictionary provided a list of all potential codes, definitions for each code, and instructions for applying codes, to standardize the coding process.

Transcripts were coded by experienced qualitative researchers trained in qualitative analysis theory and practice. Two staff members and a senior reviewer independently coded the first interview transcript from both the concept elicitation and cognitive interview phases. A post-coding comparison and reconciliation occurred, and all codes were compared, discussed, and reconciled wherever differences emerged. Once agreement between the two coders was sufficient, the remaining transcripts were each coded by one coder. A quality review by senior staff members was conducted.

The responses from the interviews were coded and analyzed by thematic code. Saturation, the point at which no substantially new themes, concepts, or terms are introduced as additional interviews are conducted, was documented by tracking concepts in a saturation grid [27, 33]. Saturation was documented in a grid with concepts listed along the y-axis and interview participants listed along the x-axis to show which concepts emerged in each interview.

## Results

### Step 1. Literature review and expert interviews

The literature review identified 26 PRO measures designed to evaluate the patient experience with treatment for type 2 diabetes. Although no instruments were identified that assessed simplicity and complexity of diabetes treatment, some questionnaires assessed concepts that may be related to treatment simplicity. For example, there are instruments with items assessing treatment satisfaction [20, 23, 24, 34, 35], satisfaction with a device [19, 23, 34], convenience/inconvenience [23, 36], burden of treatment [19–21], treatment flexibility [21, 35], convenience [20, 24], ease of use [20, 24], and perceptions of insulin therapy [34].

Two clinicians and two diabetes clinical trial outcomes experts provided input regarding aspects of treatment that may influence patient perceptions of simplicity and complexity. The four respondents suggested treatment characteristics such as dosing frequency, pill burden, need for dose titration, dose timing, food requirements, dose flexibility, and monitoring of blood sugar. Relevant concepts from the previously developed PRO measures and these expert interviews were incorporated into the concept elicitation interview guide for step 2.

### Steps 2 and 3. Concept elicitation and drafting two questionnaires

Concept elicitation interviews were conducted with patients with type 2 diabetes (N = 30; sample characteristics in Table 1). Participants reported a wide range of treatment attributes that influenced their perceptions of simplicity and complexity. See Table 2 for frequencies of patients who mentioned each concept and an example quotation for each concept. Treatment attributes often described as simple included dose timing (n = 12), preparing the medication (including the device) (n = 15), using the injection device (n = 17), and swallowing pills (n = 10). Other attributes that participants reported being simple included dose frequency (n = 3), changing the needle (n = 3), refrigeration (n = 2), and pill burden/number of pills (n = 2).

**Table 1:** Summary of Participant Characteristics

Characteristic	Concept Elicitation Interviews (N = 30)	Cognitive Interviews (N = 20)
**Age, mean years (SD)**	64.5 (11.4)	64.9 (8.3)
**Gender, n (%)** ^**1**^		
Male	16 (53.3%)	9 (45.0%)
Female	13 (43.3%)	11 (55.0%)
**Ethnicity, n (%)** ^**1**^		
Hispanic or Latino	2 (6.7%)	1 (5.0%)
Not Hispanic or Latino	26 (86.7%)	19 (95.0%)
**Race, n (%)**		
Black or African American	7 (23.3%)	6 (30.0%)
White	23 (76.7%)	14 (70.0%)
Other^2^	1 (3.3%)	--
**Employment status, n (%)**		
Full-time work	5 (16.7%)	5 (25.0%)
Part-time work	2 (6.7%)	--
Other^3^	23 (76.7%)	15 (75.0%)
**Education level, n (%)**		
University degree	9 (30.0%)	7 (35.0%)
No university degree	21 (70.0%)	13 (65.0%)
**Marital status, n (%)**		
Single	7 (23.3%)	3 (15.0%)
Married/Cohabitating/Living with partner	16 (53.3%)	12 (60.0%)
Other^4^	7 (23.3%)	5 (25.0%)
**Duration of diabetes, mean years (SD)**	18.1 (10.6)	12.9 (8.6)
**Most recent HbA1c (%), mean (SD)** ^**5**^	7.3 (1.0)	8.4 (2.5)
**BMI, mean kg/m** ^**2**^ **(SD)**	32.9 (6.4)	35.3 (8.5)
**Current treatment, n (%)**		
Oral only	3 (10.0%)	5 (25.0%)
Insulin	4 (13.3%)	2 (10.0%)
Injectable GLP-1 RA	1 (3.3%)	--
Oral and insulin	12 (40.0%)	7 (35.0%)
Oral and injectable GLP-1 RA	6 (20.0%)	2 (10.0%)
Insulin and injectable GLP-1 RA	2 (6.7%)	2 (10.0%)
Oral, insulin, and injectable GLP-1 RA	2 (6.7%)	2 (10.0%)

Abbreviations: BMI = body mass index; GLP-1 = glucagon-like peptide 1; RA = receptor agonist; SD = standard deviation

^1^Gender was missing for one concept elicitation interview participant and ethnicity was missing for two concept elicitation interview participants

^2^ Race is not mutually exclusive. One concept elicitation participant selected both “White” and “Other.”

^3^ Other employment includes retired (n = 18), disabled (n = 4), and other not specified (n = 1) for concept elicitation interviews and retired (n = 11) and disabled (n = 4) for cognitive interviews

^4^ Other marital status includes divorced (n = 5) and widowed (n = 2) for concept elicitation interviews and divorced (n = 5) for cognitive interviews

^5^ Result of most recent HbA1c test was unknown for one concept elicitation interview participant

**Table 2:** Attributes of Diabetes Medication Identified by Participants as Contributing to Treatment Simplicity or Complexity, along with Example Quotations^1^

	Simple n (%)	Example Quotation	Complex n (%)	Example Quotation
**Injection device**	17 (56.7%)	“Oh, it’s a lot easier than the vial and the needle thing. That pen makes it real easy all in all.” (M, 60 y)	3 (10.0%)	“It’s become more complicated in the sense that I’m taking more insulin now than I was earlier, and I’m taking more insulin now because I have to use the vials instead of using pens. My life would be a lot easier with insulin if I could use the insulin pens but I can’t afford that.” (M, 72 y)
**Preparing medication (including device)**	15 (50.0%)	“All you do is put a tip on it and dial it and stick it in you…That’s very simple.” (M, 56 y)	2 (6.7%)	“It just takes a longer time, the preparation of the skin with the alcohol wipes, filling the syringe with the insulin, and injecting it into your body…Taking the insulin is more complex than taking pills.” (M, 86 y)
**Dose timing (i.e., time of day)**	12 (40.0%)	“It’s simple, it is just before I go to bed” (M, 77 y)	3 (10.0%)	“I think the most difficult part is like in the morning time when I got to get it all together and do it. In the morning time is the most difficult.” (F, 69 y)
**Swallowing pill**	10 (33.3%)	“Just have a little glass of water and you swallow a pill. That’s the simplest part.” (M, 56 y)	--	
**Getting the right dose**	4 (13.3%)	“It’s simple… I don’t have to draw it. I just dial the pen.” (M, 67 y)	2 (6.7%)	“It’s on the syringe the amount that you’re supposed to take and you have to be very careful that you’re taking the right amount.” (M, 72 y)
**Food requirement**	4 (13.3%)	“Simple. None of them say with food. So, it could be either with or without.” (F, 59 y)	1 (3.3%)	“It could be a little bit complicated…you have to be alert, did you eat or you don’t eat? It will put you in a situation that you can do a mistake.” (M, 77 y)
**Flexibility of dose schedule**	4 (13.3%)	“It’s more simple for me to take it with all my medicine at once instead of once in the morning and once at night… I could either take it in the morning or at nighttime, that’s what they told me.” (F, 66 y)	--	
**Dose frequency**	3 (10.0%)	“Once a week is not bad… that’s fairly simple. There’s nothing hard about that.” (M, 73 y)	6 (20.0%)	“That’s a little bit more complicated because I take the [regular human insulin] before each meal, so we’re talking about three insulin shots a day for the [regular human insulin], so my total number of insulin shots will be five during the day.” (M, 72 y)
**Changing the needle**	3 (10.0%)	“You just screw the needle on it, so a pen and a needle, and then you just give you a shot.” (F, 66 y)	2 (6.7%)	“Well, sometimes the needles and things are difficult to hold.” (M, 76 y)
**Refrigeration**	2 (6.7%)	“When I’m always opening the refrigerator a lot, I see it and I’m reminded. So, you know, oh, let me do this. And then it’s always so in the, you know, forefront of my mind that I take this medication on time” (F, 69 y)	1 (3.3%)	“I can say it’s mostly troublesome …if I’m going on a trip, I got to make sure it’s kept cold, in a cool … Got to make sure I got a cooler or whatever. That’s the only thing I find that could be inconvenient.” (M, 67 y)
**Pill burden/Number of pills**	2 (6.7%)	“It was made easier for me by taking one pill, one in the morning and one at night rather than two in the morning and two at night, so that was made easier, just the pills being larger dosage so that I don’t have to be taking as many pills.” (M, 72 y)	--	
**Cost**	--		5 (16.7%)	“It’s become more complex…When I was first diagnosed, I was able to use the insulin pens, and so it was just a matter of maybe once a day or twice a day using the insulin pens, but now insulin is so expensive that I’ve had to go to the vials… It’s more insulin, correction insulin, that it’s become more complicated in the sense that I’m taking more insulin now than I was earlier, and I’m taking more insulin now because I have to use the vials instead of using pens. My life would be a lot easier with insulin if I could use the insulin pens but I can’t afford that.” (M, 72 y)
**Remembering to take meds**	--		4 (13.3%)	“Just remembering to do it is probably one of the hardest things.” (M, 73 y)
**Time consuming**	--		3 (10.0%)	“I can do it, it’s just that it’s burdensome…the time that it takes to get the shot ready and the amount of insulin, got it correct, monitoring throughout the day. So it’s all throughout the day it just seems like I’m doing something to monitor, and I just wish that it wouldn’t be so time-consuming and so much of what I have to do to keep my diabetes under control.” (M. 72 y)
**Bringing medication with me**	--		1 (3.3%)	“You’ve got to make sure you didn’t forget your pen. …You always fear that if you’re in a restroom and there’s people using the stalls and there’s no place to go that’s convenient where you can be by yourself… that’s primarily one of the biggest problems with taking it.” (M, 68 y)

^1^ The treatment attributes included in this table were discussed by participants in response to interview questions such as “What about your treatment is simple or easy?” “What about your treatment is complex or difficult?” “What is the most burdensome aspect of your treatment regimen?” “Since you were diagnosed, has your treatment become simpler or more complex?” and “Can you think of any other aspects of managing your diabetes that may be simple or complex?”

Treatment attributes commonly perceived as complex included dose frequency (n = 6), remembering to take medications (n = 4), cost of medications (n = 5), using the injection device (n = 3), time consuming administration procedures (n = 3), and dose timing (n = 3). Other attributes that participants reported being complex included changing the needle (n = 2), refrigeration (n = 1), food requirements (n = 1), and bringing medication with them (n = 1).

Aspects of diabetes management other than medication, such as glucose monitoring, diet, and exercise, also contributed to the overall perception of treatment simplicity or complexity. Several participants reported that the glucose monitoring process (12 participants reported this as simple and 2 reported it as complex) and finger pricking required for glucose monitoring (10 simple, 8 complex) contributed to the complexity of managing type 2 diabetes. Several participants discussed the time required for diet and exercise, along with the challenges of fitting these into their schedule. Two participants said it was simple or easy to find time for diet/exercise, while five mentioned that it was difficult. Many participants (n = 17) reported that simplifying their treatment would reduce the impact of type 2 diabetes on their lives. Quotations on the impact of treatment simplification are presented in Table 3.

**Table 3:** Example Quotations from Patients in Concept Elicitation Interviews: How Treatment Simplification Would Reduce the Impact of Treatment on Their Quality of Life ^1^

Quotation
“I could probably do a lot more activities because I wouldn’t have to worry about if it’s made simpler. I wouldn’t have the anxiety or stress, keeping track of time, I’ve got to do this and that, and it would just make it a lot simpler for me in that regard.” (M, 72 y)
“That you didn’t have to worry about taking your blood or eating exactly what you’re supposed to be eating, staying on the 45 carbs per meal, all that.” (M, 64 y)
“Not having to take four shots a day would simplify. If I only took two shots, that’s better than four. Less to keep track of.” (M, 56 y)
“I think I would travel more…it would make it more easier if I could not have to worry about my insulin…just in general not have to worry about taking it and where am I gonna be.” (F, 68 y)
“I wouldn’t have to remember each day to do it twice a day, so my life would be a heck of a lot easier if I wouldn’t have to worry about doing that every day or think about doing it.” (M, 86 y)
“You wouldn’t have to worry about finding refrigeration.” (F, 70 y)

^1^ Participants were asked “How might the impact of your diabetes treatment be reduced if treatment were simplified?”

The concepts and terms identified in these interviews were used to develop the content of the draft Sim-Q and Sim-Q-Comp. Two sets of instructions were developed for the Sim-Q so that this questionnaire could be useful in a broad range of situations. One set of instructions asks participants to assess the simplicity of a single medication for diabetes (i.e., the “single treatment version”), while the other set of instructions asks participants to consider a treatment regimen that may include multiple medications (i.e., the “full treatment regimen version”).

### Steps 4 and 5. Cognitive interviews and translatability assessment

Cognitive interviews were conducted with a total of 20 patients with type 2 diabetes (see demographic and clinical characteristics in Table 1). Each participant was asked to complete the Sim-Q (with the version of the instructions focusing on the full treatment regimen) and Sim-Q-Comp. Participants were also asked to review the alternate instructions for the Sim-Q, focusing on a single medication.

All participants reported that the Sim-Q and Sim-Q-Comp were clear and easy to complete. Still, during the interviews, it was apparent that some items required clarification to ensure that they were interpreted as intended. Therefore, minor revisions were made based on feedback from participants to clarify the instructions and individual items as necessary. The draft questionnaires were updated four times during the cognitive interviews. In total, there were four rounds of interviews (n = 10, n = 5, n = 2, n = 3), each with a slightly different version of the questionnaires.

For example, some participants had difficulty distinguishing between the items “taking the right dose” and “taking the medication at the right time.” To address this issue, “right dose” was changed to “correct dose,” and the items were presented consecutively so participants could more easily see the differences between the two questions.

The item on food requirements (originally phrased as “Food requirements [for example, some medications must be taken either with or without food]”) was revised twice during the interviews. First the phrase, “at the time you take the medication” was added to address difficulty expressed by some participants in relating the food requirements to taking the medication. The phase “or on an empty stomach” was also added to ensure this item would be applicable to diabetes medications with this requirement. These changes resulted in the revised item “Food requirements at the time you take the medication [for example, some medications must be taken either with food, without food, or on an empty stomach],” which was understood without difficulty by all participants who reviewed this final version of the item.

The instructions were revised to make the instrument more flexible for application in crossover or real-world studies (see draft instructions and items in the Supplementary Information 1). Multiple options are provided for language in the instructions, depending on the intended use of the instrument. A note was included at the end of the questionnaire to explain these options to researchers who intend to use the instrument.

A translatability assessment was conducted on the Sim-Q and Sim-Q-Comp to ensure that they are suitable for translation. The majority of the comments resulting from the translatability assessment highlighted words or phrases that may eventually present challenges for translation into some languages. For example, the item “bringing your medication with you when you need to take it away from home” may need to be rephrased as “when you need to take it outside your home/when you go out” in some languages. In these instances, alternatives can be used for translation as necessary, but no edits were recommended for the English version. Only one change was made to the Sim-Q based on the translatability assessment, the word “the” was added to “taking medication (including the steps for taking [the] tablets or giving yourself the injection)” to facilitate future translations of the item and to make it consistent with the item wording on the Sim-Q-Comp.

### Versions of the Sim-Q and Sim-Q-Comp emerging from this study

The versions of the Sim-Q and Sim-Q-Comp resulting from the cognitive interviews and translatability assessment each contain eight items assessing the simplicity/complexity of aspects of treatment, including preparing to take the medication, taking the medication at the right time, making sure you take the correct dose of medication, taking the medication, food requirements, bringing medication with you, checking blood glucose, and watching what you eat, as well as two global items assessing the simplicity/complexity of diabetes medication treatment and overall diabetes management. Both the Sim-Q and the Sim-Q-Comp ask participants to answer the items based on how they feel about their current diabetes medication “today.”

Each item of the Sim-Q is answered on a 5-point response scale, on which respondents can rate their treatment as very complex, complex, a little complex, simple, or very simple. For items where it is possible that the treatment attribute may not be relevant for all treatments or patients, the “very simple” response option allows for this possibility. For example, because some medications have no food requirements, the “very simple” response option for this item is phrased as “very simple or no food requirements.” Users of the Sim-Q can choose one of two sets of instructions, depending on which best fits the context (see draft instructions and items in the Supplementary Information 1). These instructions ask participants to assess the simplicity/complexity of either a single medication or a full treatment regimen.

Because the Sim-Q-Comp was designed to compare current treatment with previous treatment, the response options focus on this comparison. On a 5-point response scale, respondents can report how their current treatment compares to their previous treatment, with options of much more complex, a little more complex, the same, a little simpler, or much simpler.

Both the Sim-Q and Sim-Q-Comp allow for researchers to insert the names of specific medications in the instructions, so that the questionnaires can be customized for use in clinical studies focusing on specific medications.

## Discussion

Treatment for type 2 diabetes has continued to evolve over time. In addition to short- and long-acting insulins [37], the past two decades have seen the introduction of new classes of medications, such as dipeptidyl peptidase-4 inhibitors, glucagon-like peptide 1 (GLP-1) receptor agonists, sodium-glucose cotransporter-2 inhibitors, and most recently, a dual glucose-dependent insulinotropic polypeptide and GLP-1 receptor agonist [5, 38–41]. With this increasing variability in treatment options, there is the potential for both more complex and simplified treatment regimens. While combinations of treatments from multiple classes could result in more complications for patients, newer treatment options have helped simplify the process by requiring less frequent administration [2–4] or easier administration processes [11, 12]. Because these factors may have an influence on treatment adherence and treatment outcomes [15–17], it is important to understand patient perceptions of treatment simplicity and complexity. The Sim-Q and Sim-Q-Comp are the first tools designed specifically for this purpose.

In the concept elicitation interviews, patients with type 2 diabetes reported a range of treatment characteristics that contribute to the complexity of managing their diabetes, including attributes of both oral and injectable treatments. Treatment attributes that were often considered simple included dose timing, dose frequency, preparing the medication (including preparation of the injection device), using an injection device, and swallowing pills. Attributes commonly perceived as complex included dose frequency, remembering to take medications, and dose timing. Some attributes were mentioned as simple and complex by similar numbers of respondents. For example, finger pricking for glucose monitoring was perceived to be simple by 10 respondents, but complex by eight. Based on patient reports, the simplicity or complexity of a type 2 diabetes treatment regimen appears to have a meaningful impact on their lives. For example, many participants in the current study reported that simplifying treatment would alleviate worrying and stress related to diabetes treatment.

The concept elicitation results were used to generate content for two draft questionnaires, which include items that assess the treatment attributes commonly mentioned as simple or complex. The status version of the Sim-Q was developed to assess simplicity/complexity of current treatment(s), while the comparison version (Sim-Q-Comp) allows for individual patients to compare two treatments. The draft questionnaires were updated several times during the cognitive interview study based on patient feedback. In the final round of interviews with the final versions of the questionnaires, participants understood the questionnaire items as intended and were able to answer the items without difficulty.

Based on the current qualitative findings, the Sim-Q and Sim-Q-Comp appear to be clear and relevant to patients with type 2 diabetes. However, these results should be considered in the context of several limitations. First, this qualitative research represents only the first step of measure development. Future psychometric research with larger samples will be needed to derive a scoring algorithm and demonstrate reliability, validity, and ability to discriminate between treatment regimens that vary in their complexity. For example, it would be expected that the Sim-Q should distinguish between groups of patients treated with multiple daily insulin injections and patients receiving only a single weekly GLP-1 receptor agonist injection. Larger quantitative studies are needed to examine this sort of known-groups validity.

Like most qualitative research, there are also limitations associated with the sample size and level of diversity. The instrument content was derived based on input from 30 patients and then refined based on the experience of an additional 20 patients. This relatively small sample cannot be considered representative of the broader population of patients with type 2 diabetes. Furthermore, although efforts were made to recruit a sample that was reasonably diverse in terms of gender and ethnic/racial background, a sample of this size cannot provide insight into geographic, cultural, or clinical differences among patients. For example, relatively complex treatment regimens could be perceived differently by people from different cultures or by people with different levels of disease severity. The current study cannot provide insight into these potential group differences. Although qualitative coding suggests that saturation was reached and more interviews would not have identified additional content to include in the questionnaires, it is possible that interviews with a larger and broader sample could have identified other relevant medication attributes. Because of these limitations, generalizability to the broad population of people with type 2 diabetes is unknown. Accumulating Sim-Q data from future samples may help to build confidence in the instrument’s generalizability.

## Conclusions

Overall, the qualitative data from this study support the content validity of the Sim-Q and Sim-Q-Comp, and the draft questionnaires are ready for psychometric validation in larger samples of patients with type 2 diabetes. Based on patient reports, the simplicity or complexity of a treatment regimen for type 2 diabetes can have a meaningful impact on patients’ lives. As new medications are introduced and tested, it will be important to consider the extent to which these treatment options can help simplify diabetes management. The new measures introduced in this study provide a method for quantifying treatment simplicity and complexity, allowing for comparison of treatment options in clinical and observational studies.

### Electronic supplementary material

Below is the link to the electronic supplementary material.

Supplementary Material 1

## Data Availability

For permission to reproduce or use the Sim-Q or Sim-Q-Comp, please contact copyright@lilly.com. After permission is obtained, there is no fee for using these instruments.

## Abbreviations

FDA: Food and Drug Administration
PRO: Patient-reported outcome
SD: Standard deviation
Sim-Q: Simplicity of Diabetes Treatment Questionnaire
Sim-Q-Comp: Simplicity of Diabetes Treatment Questionnaire-Comparison
