# Supplementary material for: Development of a patient-reported outcome (PRO) measure to assess patient perceptions of simplicity and complexity of treatment for type 2 diabetes
Source: J Patient Rep Outcomes. 2023 Sep 6;7:89. doi: 10.1186/s41687-023-00614-7 (PMC10482816; doi:10.1186/s41687-023-00614-7)
Supplement: Supplementary file 1 — Supplementary Material 1 [file 41687_2023_614_MOESM1_ESM.docx]

# Supplemental Material

**Simplicity of Diabetes Treatment Questionnaire (Sim-Q)**

**Instructions**

- **Full treatment regimen:** Please select one response for each item to indicate how you feel today about your current treatment for diabetes. If you take more than one medication for diabetes, you should answer the questions thinking about **all** your current diabetes medications.
- **Single treatment regimen:** Please select one response for each item to indicate how you feel today about your current treatment for diabetes. If you take more than one medication for diabetes, you should answer the questions thinking about **only one** of the medications.

Please complete this questionnaire thinking about: ______________________
 *Name of medication*

**Items**

**Item stem:** Some aspects of diabetes treatment are simple, and others are complex. **How simple or complex are the following aspects of your current diabetes treatment?**

1. **Preparing** to take the medication (for example, preparing an injection device or getting water for taking tablets)
2. Taking the medication at the **right time**
3. Making sure you take the **correct dose** of medication each time you take it
4. **Taking** the medication (including the steps for taking the tablets or giving yourself the injection)
5. **Food requirements** at the time you take the medication (for example, some medications must be taken either with food, without food, or on an empty stomach)
6. **Bringing medication** with you when you need to take it away from home
7. **Checking your blood glucose** levels on your own
8. **Watching what you** **eat** (for example, eating the right foods and avoiding some foods)

**Please answer the following questions about your diabetes treatment:**

1. **How simple or complex is your** **medication treatment** for diabetes?
2. **Overall, how simple or complex is it to manage your diabetes**, including medication, checking your blood glucose levels, diet, and any other aspects of diabetes treatment?

**Simplicity of Diabetes Treatment Questionnaire - Comparison (Sim-Q-Comp)** **Instructions**

- You have been receiving **[*medication for diabetes*]**. When completing the questions below, please compare **[your *current diabetes medication*]** to **[*your previous medication*]**.

Please select one response for each item to indicate how you feel today about your current treatment compared to your previous treatment.

**Items**

Item Stem: Some aspects of diabetes treatment are simple, and others are complex. Compared to your previous treatment, **how simple or complex are the following aspects of your current treatment?**

**Compared to my previous treatment, my current treatment is…**

1. **Preparing** to take the medication (for example, preparing an injection device or getting water for taking tablets)
2. Taking the medication at the **right time**
3. Making sure you take the **correct dose** of medication each time you take it
4. **Taking** the medication (including the steps for taking the tablets or giving yourself the injection)
5. **Food requirements** at the time you take the medication (for example, some medications must be taken either with or without food)
6. **Bringing medication** with you when you need to take it away from home
7. **Checking your blood glucose** levels on your own
8. **Watching what you eat** (for example, eating the right foods and avoiding some foods)

**Please answer the following questions about your diabetes treatment:**

1. Compared to **[*your previous medication*]**, **how simple or complex is your [*current medication treatment for diabetes]***?
2. **Overall**, compared to the time before you started **[*your current medication*]**, **how simple or complex is it to manage your diabetes**, including medication, checking your blood glucose levels, diet, and any other aspects of diabetes treatment?

Note to users of this questionnaire: Italicized terms in brackets may be customized based on the needs of the specific study, trial, or clinical situation. For example, you may include names of specific medications or phrases such as “your current medication,” “the study medication,” “the medication you are receiving in this study,” “your previous medication for type 2 diabetes,” or “your treatment before you started this study.”
